# Supplementary material for: Multi-dimensional Precision Livestock Farming: a potential toolbox for sustainable rangeland management
Source: PeerJ. 2018 May 30;6:e4867. doi: 10.7717/peerj.4867 (PMC5984589; doi:10.7717/peerj.4867)
Supplement: Supplemental Information S1 [file peerj-06-4867-s001.docx]

# Appendix S1

Data was obtained at Fortín Chacabuco ranch (41° 0' 46.36" S, 71° 8' 35.78" W) that belongs to the pre-mountain ecological area located in northern Patagonia. Data from tags were collected in November 2014. During that period animals were kept in a ~90 ha paddock.

## Paddock description:


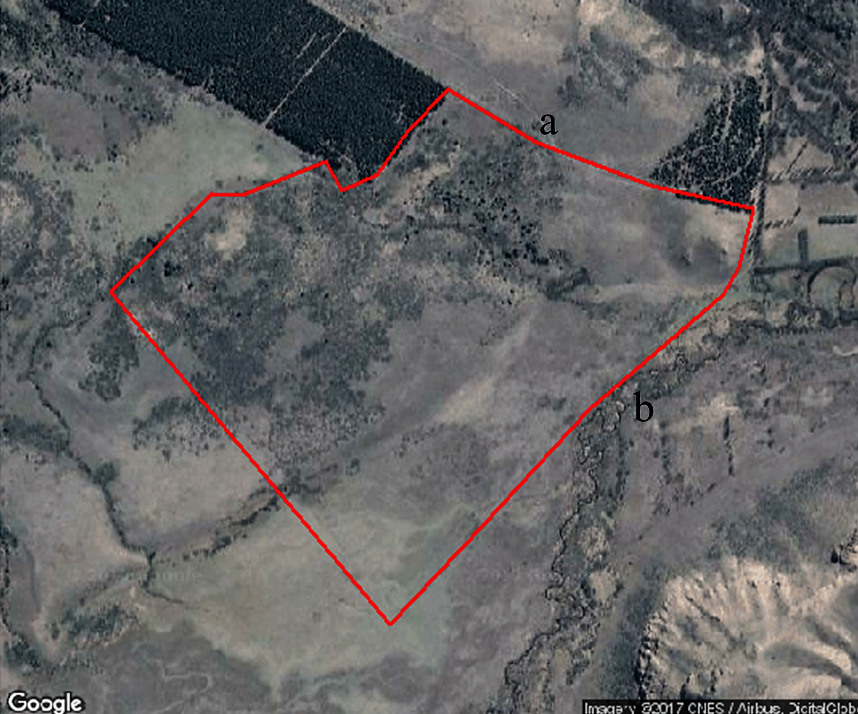


Figure S1.1: Google Earth image (Google Earth Pro Version 7.3.0.3832 32-bit, September 30, 2017, Neuquén, Argentina. Digital Globe 2017. <http://www.earth.google.com>) to show the heterogeneity of resources distribution. Letters a and b are the points from where pictures of Figure 2 were taken. The red solid line represents the paddock’s fence.


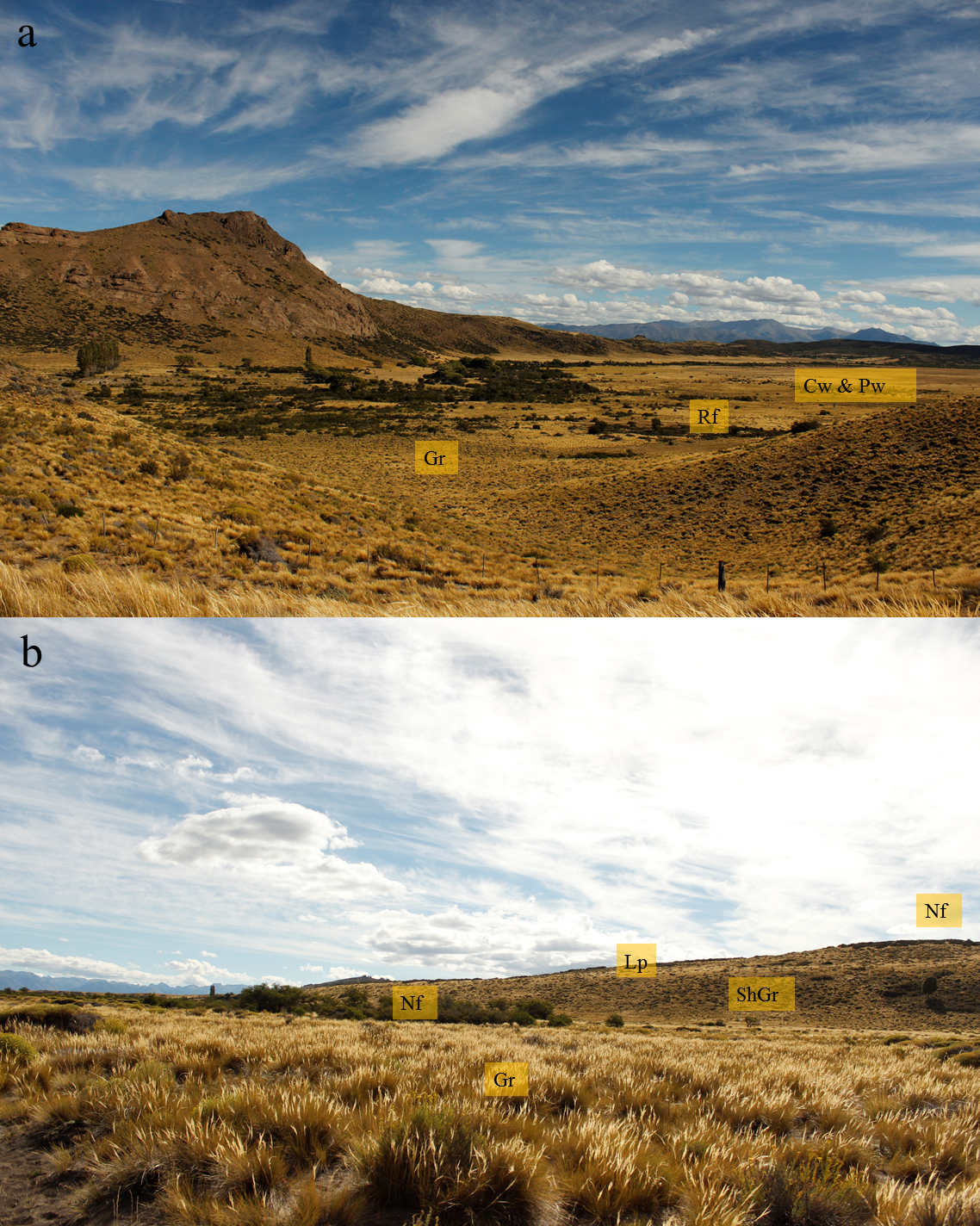


Figure S1.2: Photographs (by Agustina di Virgilio) that show with more detail vegetation units in this paddock. Cw and Pw indicate Central and Peripheral wetlands (meadows or *mallines*) respectively; Gr and ShGr are Grasslands and Shrubland-Grasslands respectively; Lp represents Low production areas; and Rf and Nf are Riparian and Native forests.

## Elevation and Slope maps:


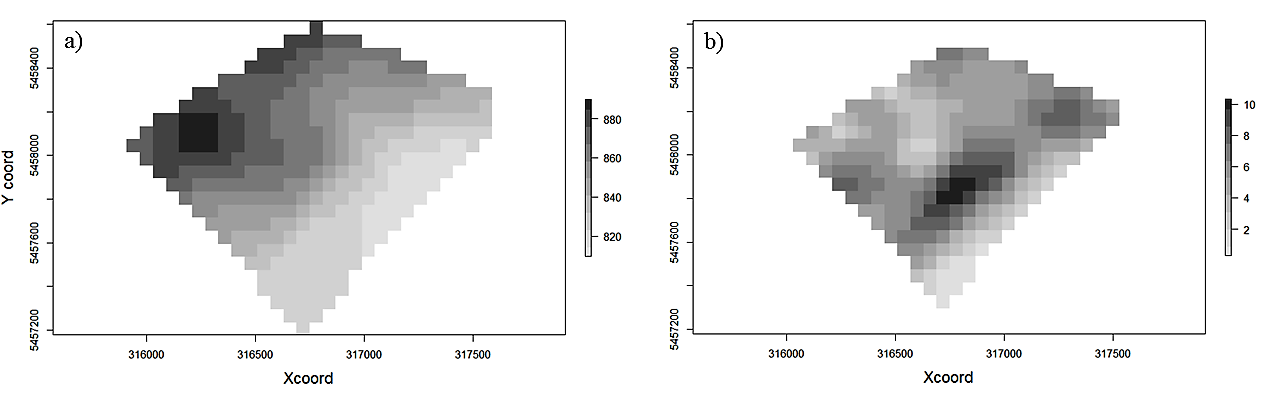


Figure S1.3: Digital Elevation Model (DEM) from the paddock ([www.earthexplorer.usgs.gov](http://www.earthexplorer.usgs.gov), resolution 60 x 60 m). In the X and Y axis are expressed the projected coordinates in meters (UTM 19 South projection). The elevation (a) of this paddock ranges between 800 and 900 meters above sea level; and the slopes (b) ranges between 0 and 10 degrees. Darker colours indicate higher values of elevation and slope. This information was extracted using the functions raster () and extract from R package raster (Hijmans, 2016), and slopeasp() from package landsat (Goslee, 2011).

## Risk map:


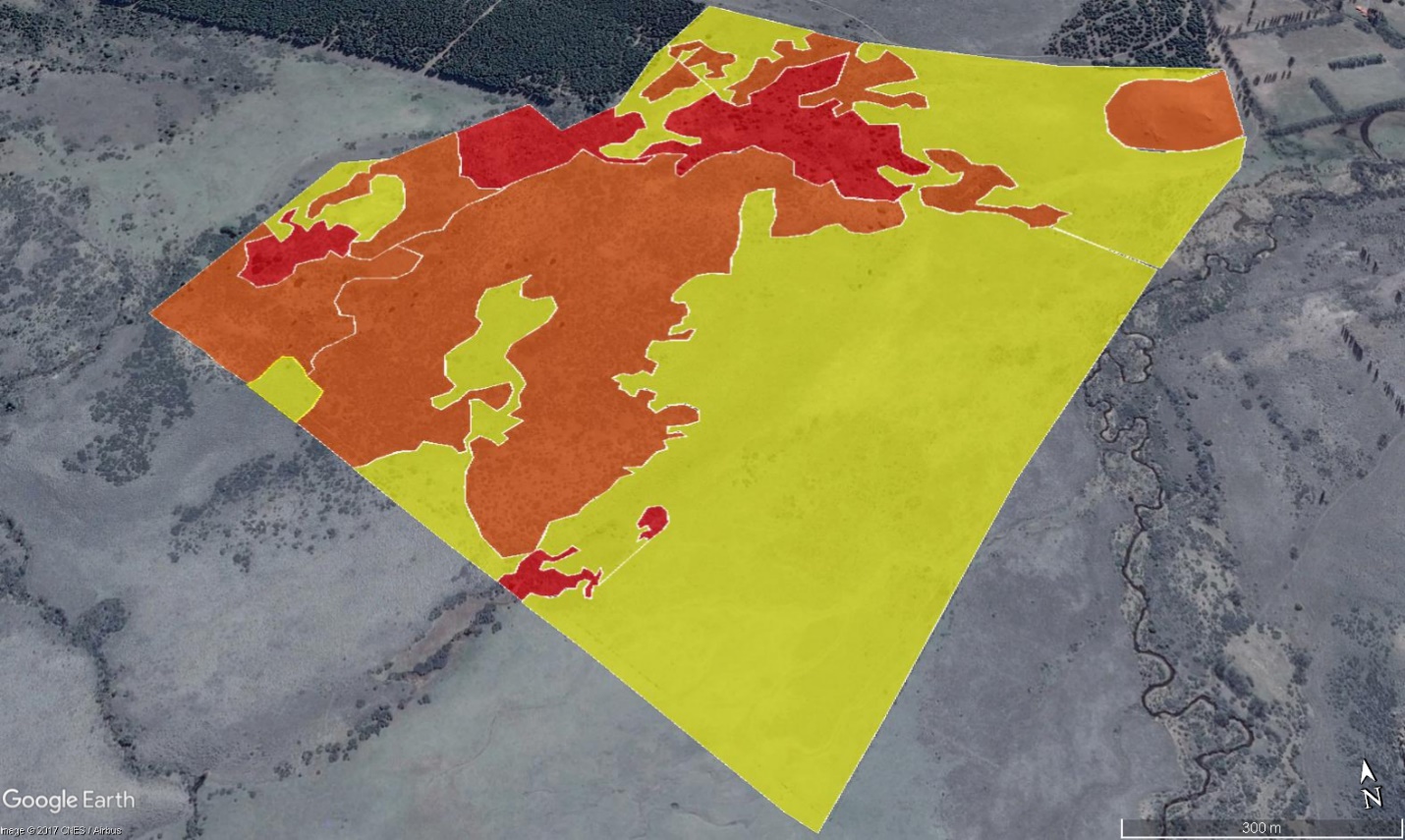


Figure S1.4: Risk Map constructed with information about vegetation type and closeness, GPS locations of predators’ feces and paw prints, and GPS locations of prey carcasses including livestock and wild herbivores. Particularly for this area, the main predators are pumas and foxes, and the potential wild preys are guanacos, exotic red deer and European hares. Areas colored in red are high risk zones, in orange are medium risk level zones, and in yellow are representing the low risk level zones. This map was constructed by first, generating a density kernel distribution using GPS coordinates, and estimating the contour of 95, 75 and 50%, to construct low, medium and high-risk areas. Inside each of these contours, the risk level was weighted according vegetation type: closed vegetation, such as riparian and native forest, increased the level of perceived risk, and open areas, such as wetlands and grassland, decreased the perceived predation risk. All this processing was performed using R software (R Core Team, 2016). We used the function writeOGR() and readOGR() from rgdal package (Bivand, Keitt & Rowlingson, 2016) to create and read shape files, and the SpatialPoints() function from sp package (Pebesma & Bivand, 2005) to convert GPS data into an spatial object. Then, we used the functions kernelUD() and getvericeshr() from adehabitatHR package (Calenge, 2006) to estimate the density kernel and the contours, respectively.

## Intra-specific competition map:


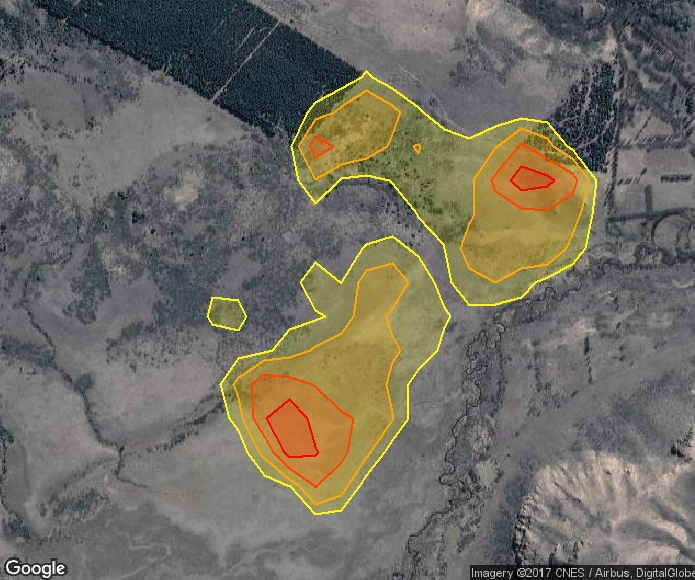


Figure S1.5: Competition map constructed with diurnal GPS data from 20 individuals from the same flock than the DD-tagged individuals during approximately 4 months. Due to sheep grazing occurs mainly during daytime, we used diurnal locations was made to infer space use patterns of grazing animals. Areas colored in red were used with more intensity by the flock (i.e., which correspond to higher intra-specific competition areas), in light and dark orange are medium competition level zones, and in yellow and yellow are representing the low competition level zones. Data processing was performed using R software (R Core Team, 2016). We used the function writeOGR() and readOGR() from rgdal package (Bivand, Keitt & Rowlingson, 2016) to create and read shape files, and the SpatialPoints() function from sp package (Pebesma & Bivand, 2005) to convert GPS data into an spatial object. Then, we used the functions kernelUD() and getvericeshr() from adehabitatHR package (Calenge, 2006) to estimate the density kernel and the contours, respectively. The competition levels corresponded to the values of the contours, which indicate the intensity of use of the different regions inside the paddock (high contour values indicate lower intra-specific competition levels and lower contour values indicate higher levels of intra-specific competition).

## Multi-sensor tags on sheep:


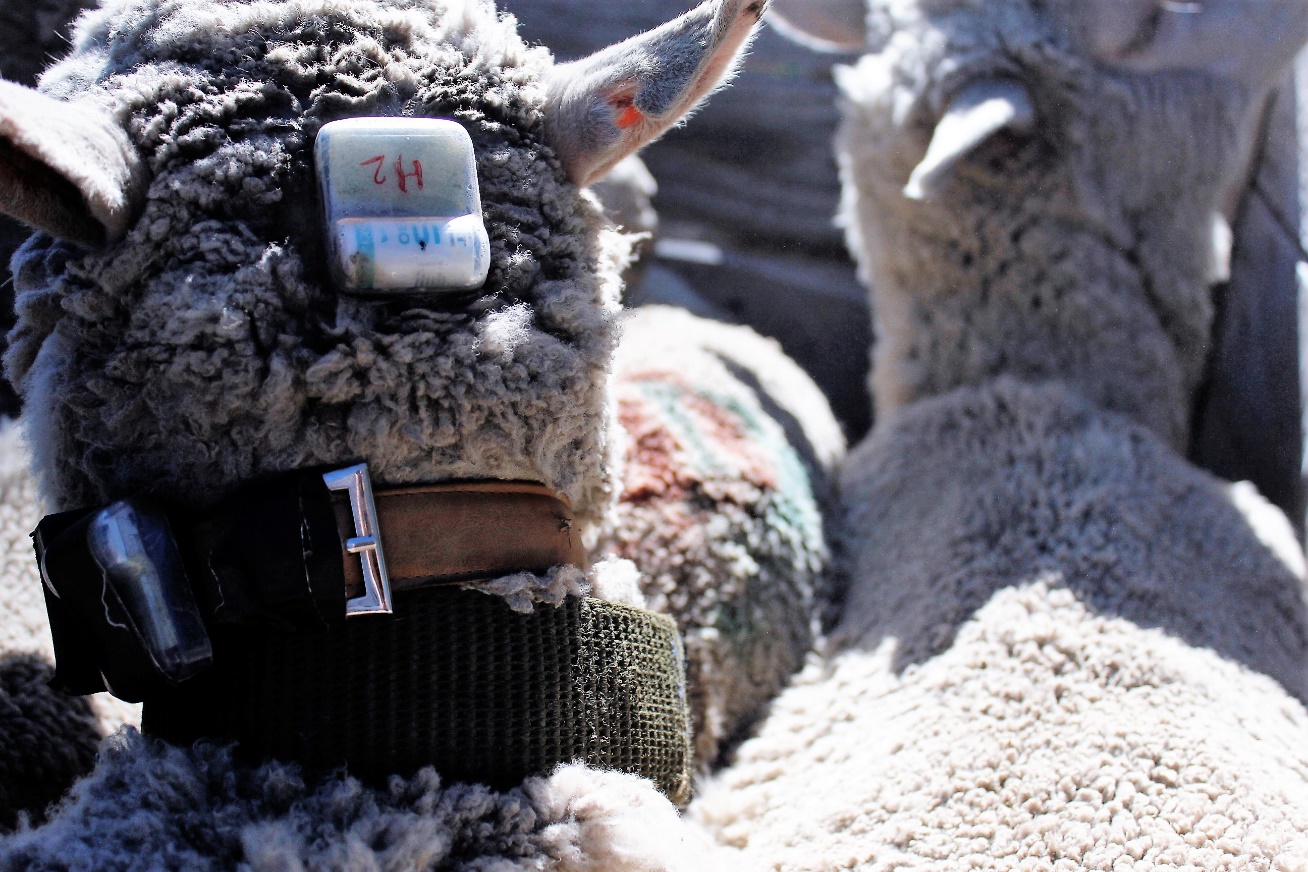


Figure S1.6: Merino sheep equipped with two Daily Diaries (one on the back of the head, and the other on the neck) and a GPS device (Photo by: Juan Manuel Morales).

## References

Bivand RS., Keitt T., Rowlingson B. 2016. rgdal: Bindings for the Geospatial Data Abstraction Library.

Calenge C. 2006. The package adehabitat for the R software: a tool for the analysis of space and habitat use by animals. *Ecological Modelling* 197:516–519.

Goslee SC. 2011. Analyzing Remote Sensing Data in R: The landsar Package. *Journal of Statistical Software* 43:1–25.

Hijmans RJ. 2016. Geographic Data Analysis and Modeling.

Pebesma EJ., Bivand RS. 2005. Classes and methods for spatial data in R. *R News* 5.

R Core Team. 2016. R: A language and envrionment for statistical computing.
