# Supplementary material for: Multi-dimensional Precision Livestock Farming: a potential toolbox for sustainable rangeland management
Source: PeerJ. 2018 May 30;6:e4867. doi: 10.7717/peerj.4867 (PMC5984589; doi:10.7717/peerj.4867)
Supplement: Supplemental Information S2 [file peerj-06-4867-s002.docx]

Appendix S2

## Observed behaviors:

Table S2.1: Description of each behavior observed during training data collection.

| Behavior | Description |
| --- | --- |
| Grazing | The sheep is standing up, with the head down or inclined towards the vegetation. The overall movement of the body is absent or it is very slow. |
| Bite | Inside each grazing period, the sheep clip the vegetation moving their head sharply forward or backward. |
| Search | This behavior is characterized by sheep rising the head and performing lateral movements. Also, during the search the overall movement of the body was higher than during grazing. |
| Resting-Ruminating | The sheep lies on the grass, with the head mainly up, but scanning around. Depending on the location of the sheep, the jaw movement associated to rumination can be detected but most of the times the observer was not able to differentiate between resting and ruminating behaviors. Also, both behaviors can be performed simultaneously by sheep, that is why we referred to these behaviors combined. |
| Vigilance | It was defined by the sheep rising the head and point it towards a particular direction, maintaining it for a certain amount of time. This behavior has a variable length, sometimes can be really short, and other times can last for several seconds. |
| Fast Walk | It was characterized by a fast displacement of the animal (running) |

## Behavior classification


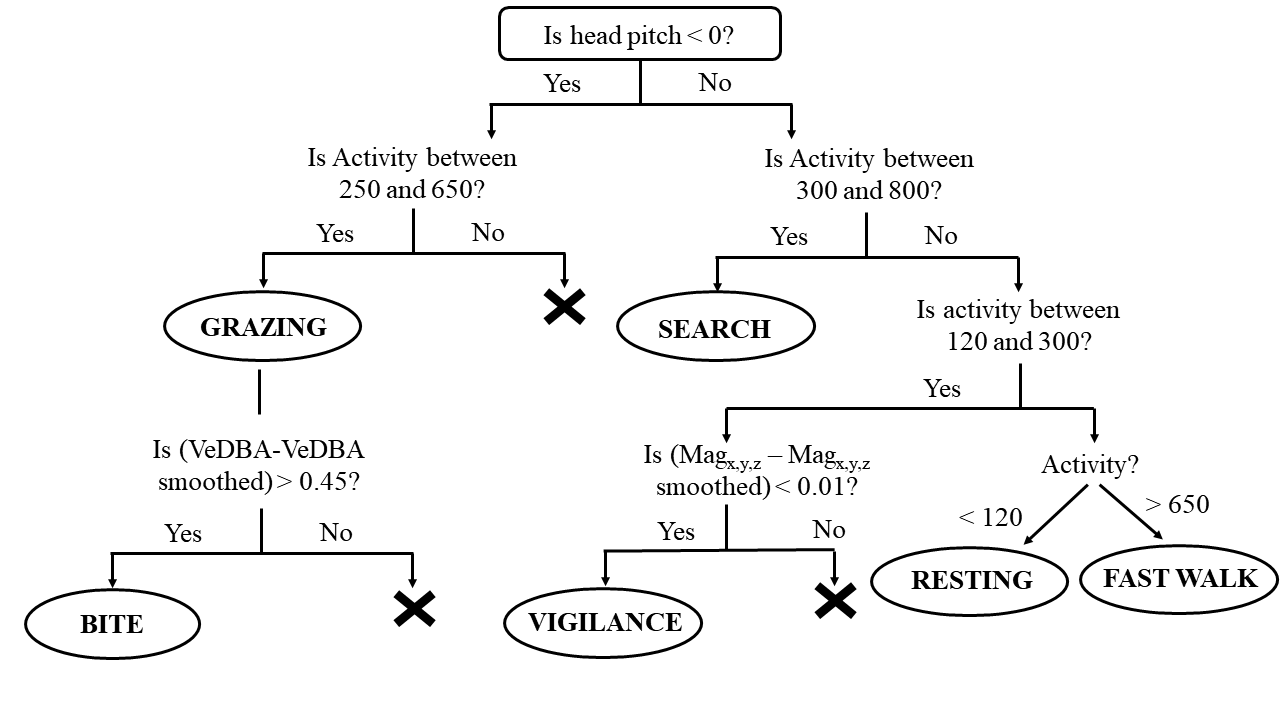


Figure S2.1: Classification tree for all behaviors considered in this work: Grazing, Search, Bite, Vigilance, Resting and Fast Walk. Each threshold value from the tree was obtained from the proportion of data from the training data set selected to construct the tree. The rest of the data was used to apply this tree and to estimate classification accuracy.

Table S2.2: Classification accuracy for each behavior obtained from the two DD location attachments (i.e., head and collar).

| Attachment location | Grazing | Search | Fast Walk | Vigilance | Resting | Bites |
| --- | --- | --- | --- | --- | --- | --- |
| Head DD | 93% | 87% | 97% | 79% | 86% | 100% |
| Collar DD | 87% | 36% | 95% | 66% | 82% | 89% |

Table S2.3: Confusion matrix

We build a confusion matrix for both attachment places (head, table 2.1; collar, table 2.2) counting the number of observations from each behavior that were classified as the observed behavior (diagonal with numbers in bold), and the number of observations that were classified as a different behavior.

Table S2.3.1: Confusion matrix for head DD data. Percentage values are expressed within brackets. Precision values (True Positive / [True Positive + False Positive]) for Grazing, Searching, Walking, Vigilance and Resting were: 0.99, 0.25, 0.73, 0.16 and 0.98, respectively. Recall values (True Positive / [True Positive + False Negative]) for Grazing, Searching, Walking, Vigilance and Resting were: 0.96, 0.89, 0.98, 0.80 and 0.92, respectively.

|  | Observed behaviors | | | | |
| --- | --- | --- | --- | --- | --- |
| Head DD | Grazing | Searching | Walking | Vigilance | Resting |
| Grazing | **13427 [93%]** | 10 [3%] | 0 [0%] | 0 [0%] | 0 [0%] |
| Searching | 922 [6%] | **310 [89%]** | 2 [2%] | 0 [0%] | 0 [0%] |
| Walking | 0 [0%] | 30 [8%] | **82 [98%]** | 0 [0%] | 0 [0%] |
| Vigilance | 0 [0%] | 0 [0%] | 0 [0%] | **41 [80%]** | 650 [8%] |
| Resting | 137 [1%] | 0 [0%] | 0 [0%] | 10 [20%] | **7221 [92%]** |

Table S2.3.2: Confusion matrix for collar DD data. Percentage values are expressed within brackets. Precision values (True Positive / [True Positive + False Positive]) for Grazing, Searching, Walking, Vigilance and Resting were: 0.92, 0.67, 0.44, 0.12 and 0.99, respectively. Recall values (True Positive / [True Positive + False Negative]) for Grazing, Searching, Walking, Vigilance and Resting were: 0.99, 0.85, 0.95, 0.72 and 0.79, respectively.

|  | Observed behaviors | | | | |
| --- | --- | --- | --- | --- | --- |
| Collar DD | Grazing | Searching | Walking | Vigilance | Resting |
| Grazing | **12601 [93%]** | 93 [26%] | 0 [0%] | 0 [0%] | 0 [0%] |
| Searching | 967 [6.7%] | **135 [39%]** | 4 [5%] | 0 [0%] | 20 [0.2%] |
| Walking | 0 [0%] | 101 [29%] | **80 [95%]** | 0 [0%] | 0 [0%] |
| Vigilance | 0 [0%] | 21 [6%] | 0 [0%] | **37 [72%]** | 1607 [20%] |
| Resting | 37 [0.3%] | 0 [0%] | 0 [0%] | 14 [28%] | **6244 [79.8%]** |
