# Supplementary material for: Multi-dimensional Precision Livestock Farming: a potential toolbox for sustainable rangeland management
Source: PeerJ. 2018 May 30;6:e4867. doi: 10.7717/peerj.4867 (PMC5984589; doi:10.7717/peerj.4867)
Supplement: Supplemental Information S3 [file peerj-06-4867-s003.docx]

# Appendix S3

## Dead Reckoning Paths correction:


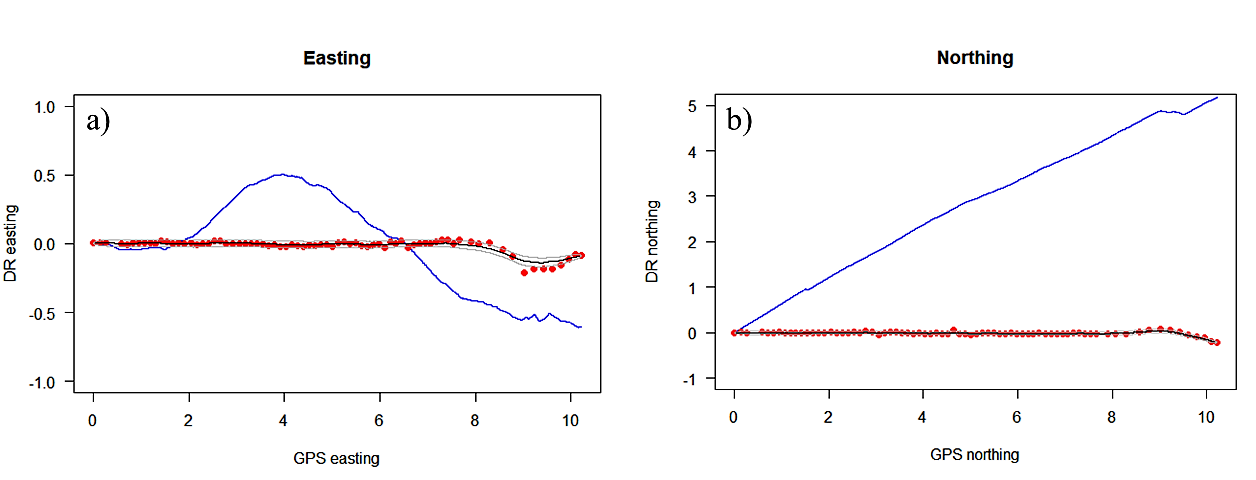


Figure S3.1: Decomposition in easting (a) and northing (b) dimensions for a 7 hours fragment of the Dead Reckoned path (blue line), the GPS locations (red dots), and the Bayesian Melding corrected path (black line) and the 95% confidence intervals (gray lines). As is shown in the figure, the DR path accumulates errors in the estimation of locations (the blue line is very distant from GPS data); while the corrected path (black line) fits accurately to GPS data.

## Example code:

#Step 1 - Upload packages####

library("TrackReconstruction")

library("BayesianAnimalTracker")

#Step 2 - Upload DD the data####

demo.data <- read.delim("demo_data5.csv", header = T, sep = ",")

#Step 3 - Estimate DR path####

#3.1- Estimate the betas:

betas <- Standardize(1, 1, 1, -1, -1, 1, min(demo.data$MagSurge), max(demo.data$MagSurge), min(demo.data$MagHeave), max(demo.data$MagHeave), min(demo.data$MagSway), max(demo.data$MagSway), min(demo.data$AccSurge), max(demo.data$AccSurge), min(demo.data$AccHeave), max(demo.data$AccHeave), min(demo.data$AccSway), max(demo.data$AccSway))

#3.2- Estimate the Declination and Inclination of magnetic field.

# extracted from: http://www.geomag.bgs.ac.uk/data_service/models_compass/wmm_calc.html

D_MF <- 5.589 #Main Field Declination (in degrees east)

I_MF <- -42.836 #Main Field Inclination (in degrees east)

#3.3- Convert Date and Time data from DD into DateTime POSIXct format:

Sys.setlocale("LC_TIME", "C")

demo.data$DateTime <- as.POSIXct(strptime(paste(demo.data$Date, demo.data$Time), "%d/%m/%Y %H:%M:%S"))

#3.4- Define speed (in this case, obtained from GPS data):

speed <- 0.5

#3.5- Generation of DR patch using the DeadReckoning function from "TrackReconstruction" package:

DRoutput <- DeadReckoning(demo.data, betas, decinc = c(D_MF, I_MF), Hz = 40, RmL = 2, DepthHz = 1, SpdCalc = 3, MaxSpd = speed)

#Step 4 - Bayesian Melding GPS correction of DR path####

#4.1- Upload GPS data (in this case, 1 min resolution):

gps.H1 <- read.delim("H1_GPS_DEMO1min.txt", header = T)

#4.2- Convert datetime data from GPS into DateTime POSIXct format:

gps.H1$DateTime <- as.POSIXct(strptime(gps.H1$datetime, "%d/%m/%Y %H:%M:%S"))

#4.3- Matching time of the GPS and DR:

gpsdata <- gps.H1[gps.H1$DateTime %in% DRoutput$DateTime, ]

#4.4- Format GPS data using the GPStable function:

gpsformat <- GPStable(gpsdata)

#4.5- Define starting and ending points from GPS data:

K.demo <- nrow(gpsformat)

DRstart <- min(which(DRoutput$DateTime==gpsformat$DateTime[1]))

DRend <- max(which(DRoutput$DateTime==gpsformat$DateTime[K.demo]))

#4.6- Thin the data (Original 40Hz, for now only working with 1Hz):

DRworking <- DRoutput[c(DRstart:DRend)[c(DRstart:DRend)%%40==1], ]

#4.7- Calculate the northing in km for GPS data and for DR paths:

T.demo <- nrow(gpsformat)

GPSnorthing <- c(cumsum(gpsformat$DistanceKm[-1]*cos(gpsformat$BearingRad[-T.demo])))

GPSeasting <- c(cumsum(gpsformat$DistanceKm[-1]*sin(gpsformat$BearingRad[-T.demo])))

#Original unit of DR is in meters, so we convert it in kilometers:

DRnorthing <- (DRworking$Ydim - DRworking$Ydim[1])/1000

DReasting <- (DRworking$Xdim - DRworking$Xdim[1])/1000

#4.8- Bayesian Melding calculation for northing and easting:

nlist <- as.dataList(DRnorthing, GPSnorthing, Ytime = format(gpsformat$DateTime, "%d-%b-%Y %H:%M:%S"), Xtime = format(DRworking$DateTime, "%d-%b-%Y %H:%M:%S"), s2G=0.0001, timeUnit = 40*60, betaOrder=1)

npost <- BMAnimalTrack(nlist, BMControl(print=TRUE, returnParam=TRUE))

elist <- as.dataList(DReasting, GPSeasting, Ytime = format(gpsformat$DateTime, "%d-%b-%Y %H:%M:%S"), Xtime = format(DRworking$DateTime, "%d-%b-%Y %H:%M:%S"), s2G=0.0001, timeUnit = 40*60, betaOrder = 1)

epost <- BMAnimalTrack(elist, BMControl(print = TRUE, returnParam = TRUE))

#Step 5 - Plotting DR GPS-corrected paths####

cPathInKM <- cbind(epost$etaMar[,1], npost$etaMar[,1])

cPathInDeg <- KMToDeg(cPathInKM, gpsformat [1, c(3, 2)])

plot(cPathInDeg[, ], type="l", lwd=2) #DR corrected path

points(gpsformat [, c(3, 2)], col="red", pch=16) #GPS points

# lines(gpsformat [, c(3, 2)], col="blue", lwd = 2, lty = 2) #GPS path
