# Supplementary material for: Multi-dimensional Precision Livestock Farming: a potential toolbox for sustainable rangeland management
Source: PeerJ. 2018 May 30;6:e4867. doi: 10.7717/peerj.4867 (PMC5984589; doi:10.7717/peerj.4867)
Supplement: Supplemental Information S4 [file peerj-06-4867-s004.docx]

# Appendix S4


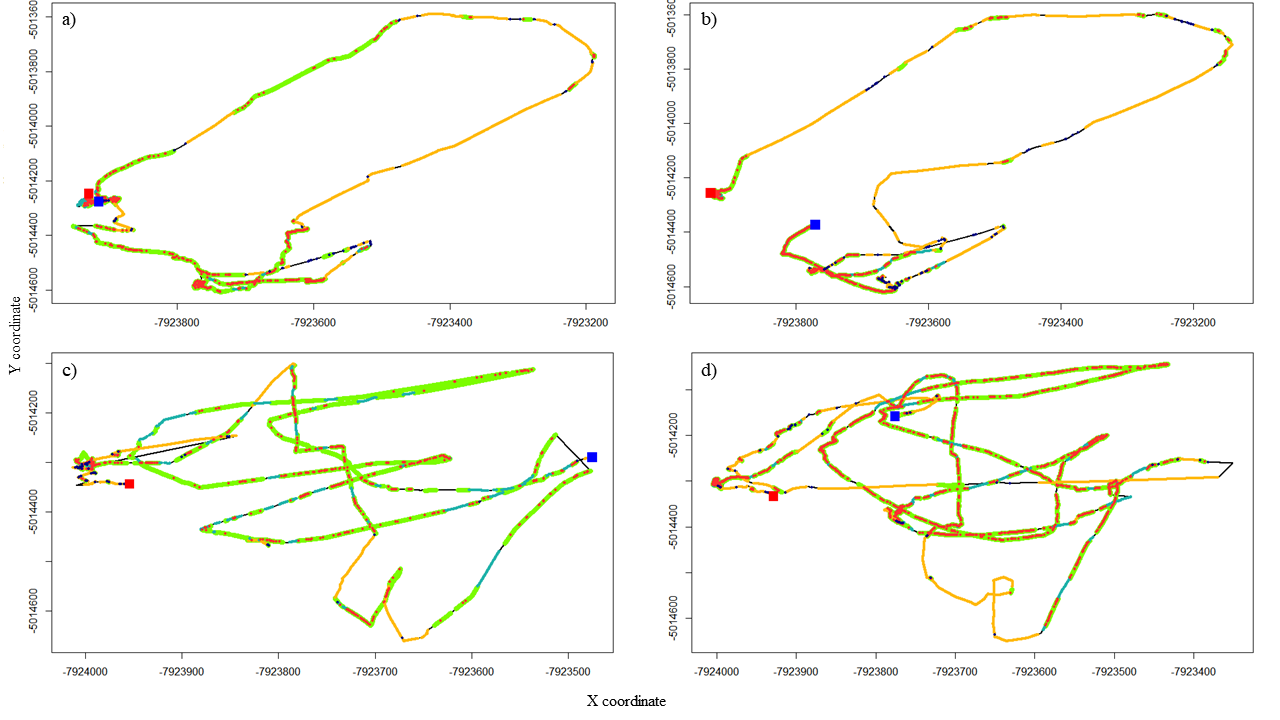


Figure S4.1: GPS-corrected dead-reckoned path showing space-associated behaviors from sheep over 24 hours. The red and blue squares represent the start and end points of the trajectory. Grazing points are colored in green, resting in orange, search in red, fast walk in light blue and vigilance in blue. The uncolored parts of the paths (black line) are location that could be classified in any of the behaviors considered in this work. Panels a and c correspond to one sheep and panels c and d to a different individual. The upper panels (a and b) are the movement path of two different sheep of one day, and lower panels are movement paths of those individuals from a different day.


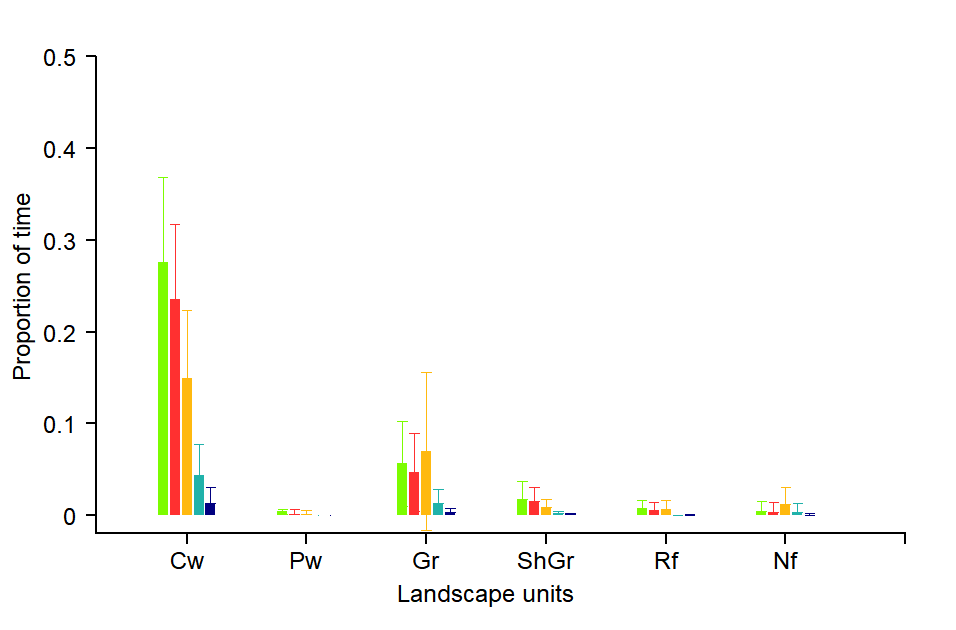


Figure S4.2: Proportion of time spent in each behavior in all vegetation units present in the paddock. Each bar represents the mean proportion of time (plus standard deviation error lines) for each behavior in each vegetation unit. Green represents the time spent grazing, red represents search, orange - resting, light blue - fast walk and navy blue - vigilance. The vegetation units used by sheep were; central wetlands (Cw), peripheral wetlands (Pw). grasslands (Gr), shrubland-grassland (ShGr), riparian forest (Rf), and native forest (Nf).

Figure S4.3: In all the following pictures (D3.1, D3.2 and D3.3), panels a and c are the reconstructed movement path of two different sheep within the paddock (delineated by red lines). This path was color-coded according to estimated bite rate: The white solid line in the movement path shows parts of the trajectory where grazing did not occur while different tones of green represent different bite rates. Light green tones represent low bite rates (between 1 and 2 bites per second), and darker tones represent higher bite rates (commonly between 5 and 7 bites per second). In panels b and d are shown the cumulative bites over time through the day for the same period of panels a and c, respectively. The red dashed line indicates midnight, and the horizontal blue dashed lines for sheep 1, represent the maximum number of bites observed for the sheep 2.


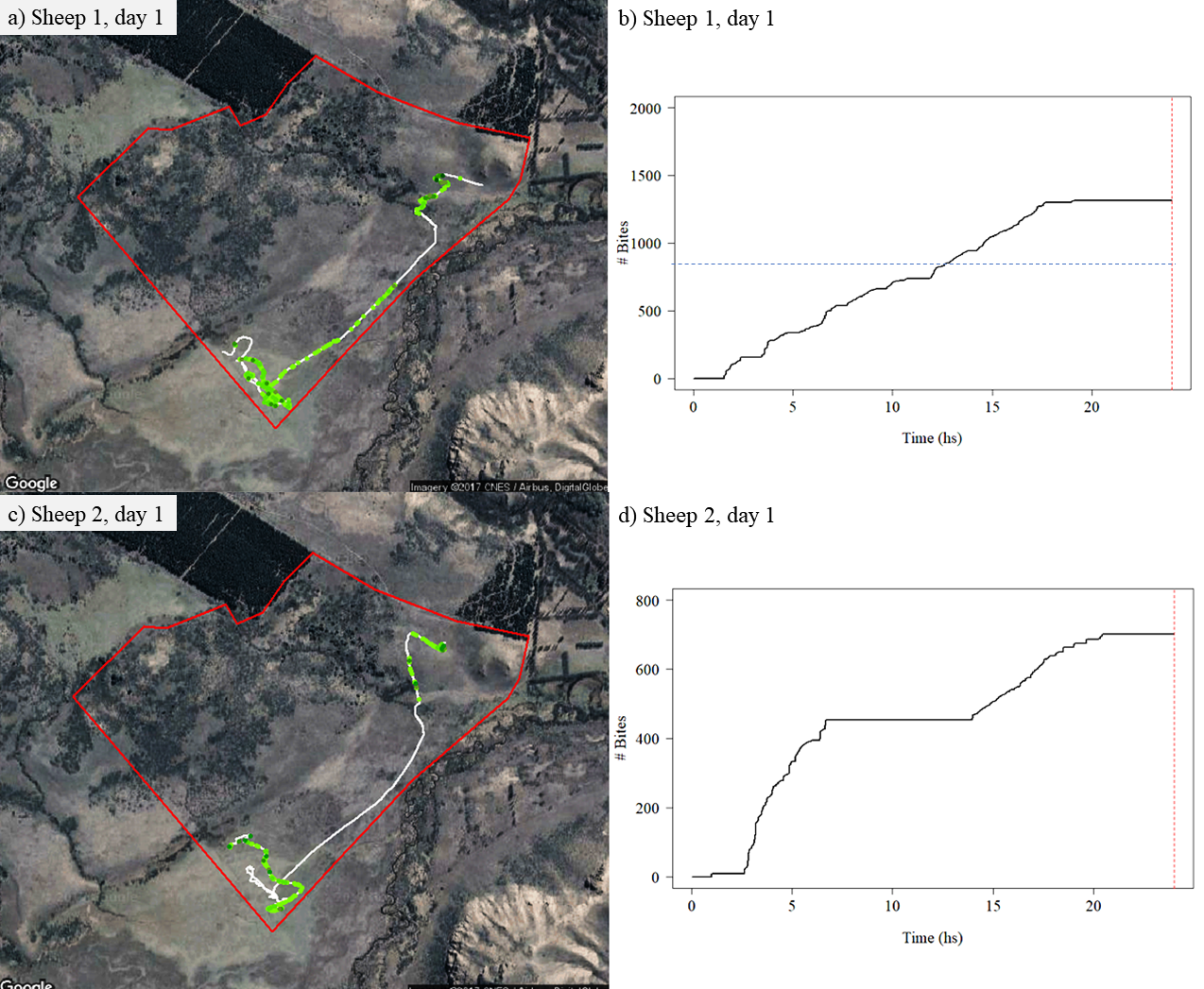


Figure S4.3.1: Movement path with bite rate (a and c) and accumulated bites through time (b and d) for sheep 1 and sheep 2, respectively on day 1.


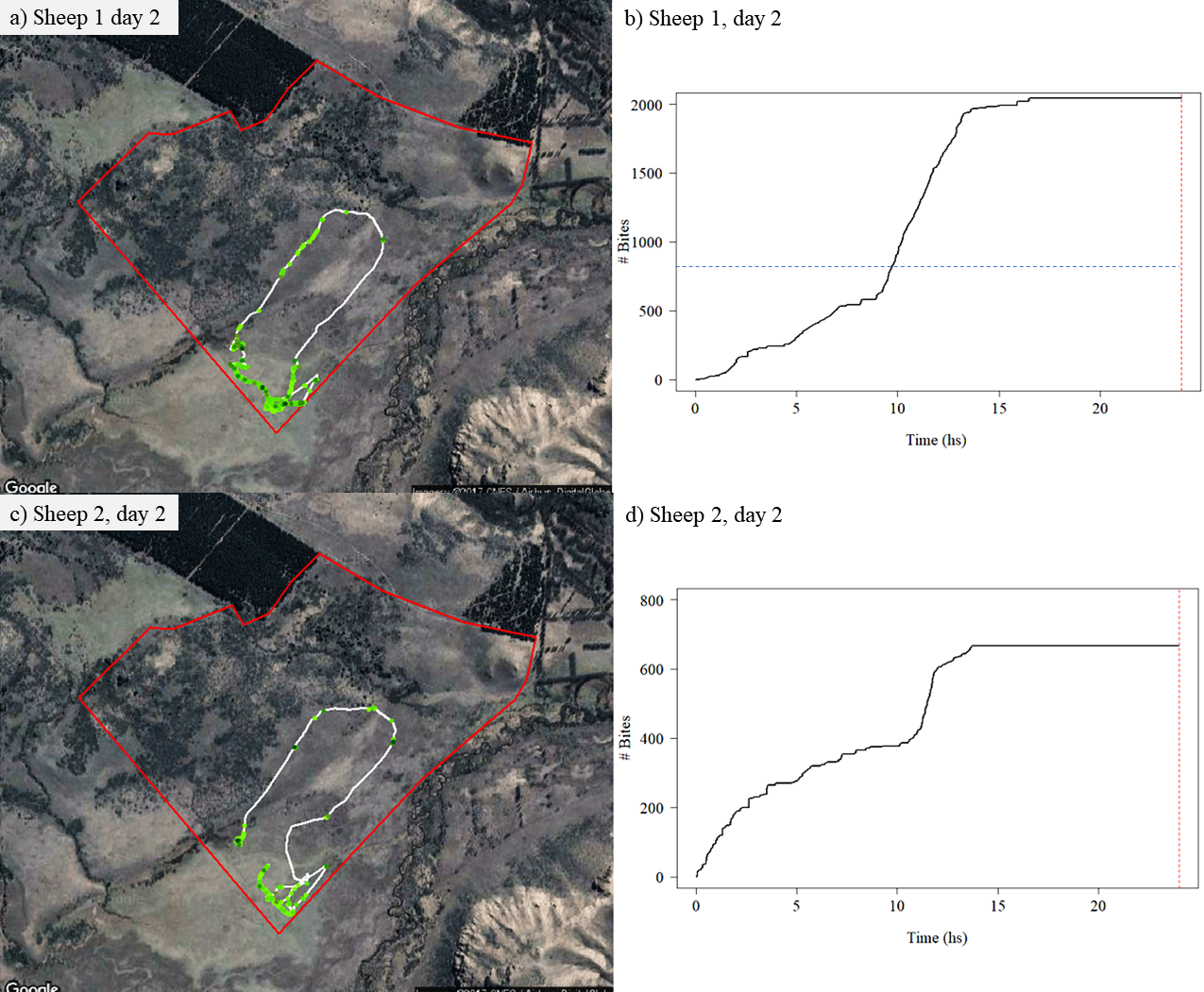


Figure S4.3.2: Movement path with bite rate (a and c) and accumulated bites through time (b and d) for sheep 1 and sheep 2, respectively on day 2.


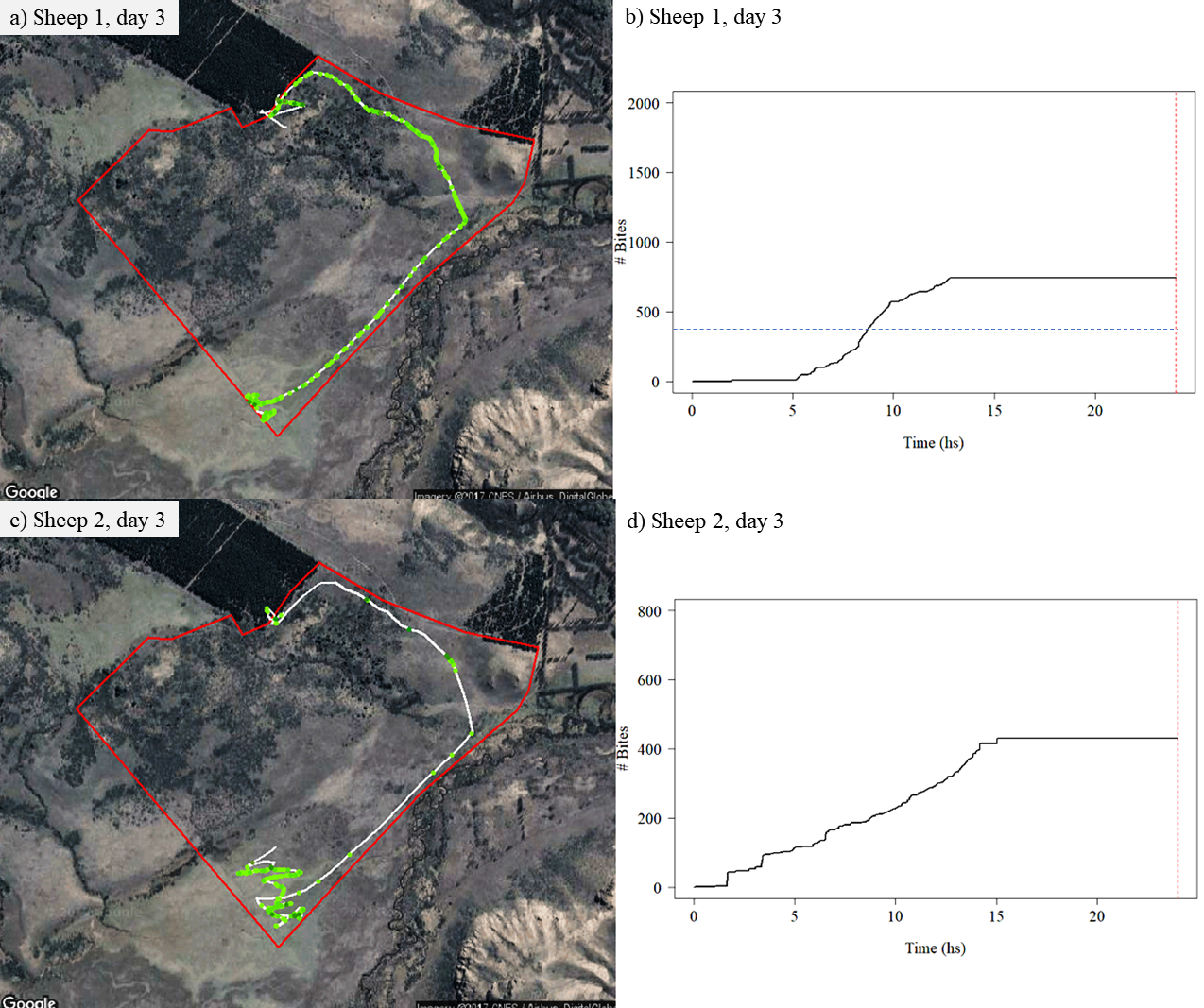


Figure S4.3.3: Movement path with bite rate (a and c) and accumulated bites through time (b and d) for sheep 1 and sheep 2, respectively on day 3.

Figure S4.4: In all the following pictures (D3.1, D3.2 and D3.3), panels a and c are the reconstructed movement path over 24 hours of two different sheep within the paddock (delineated by red lines). This path was color-coded according to estimated vigilance levels: The white solid line in the movement path shows parts of the trajectory where vigilance did not occur while different tones of blue represent different levels of vigilance. Light blue tones represent low vigilance levels and darker tones represent higher vigilance levels. In panels b and d are shown the cumulative vigilance interruption over time through the day for the same period of panels a and c, respectively. The red dashed line indicates midnight, the horizontal blue dashed lines represent the maximum number of bites observed in the other sheep on the same day, and the horizontal black dashed lined indicates the maximum value of vigilance observed for sheep 1 (to have an idea of the magnitude of vigilance between both individuals, not only daily but for the entire sampling period).


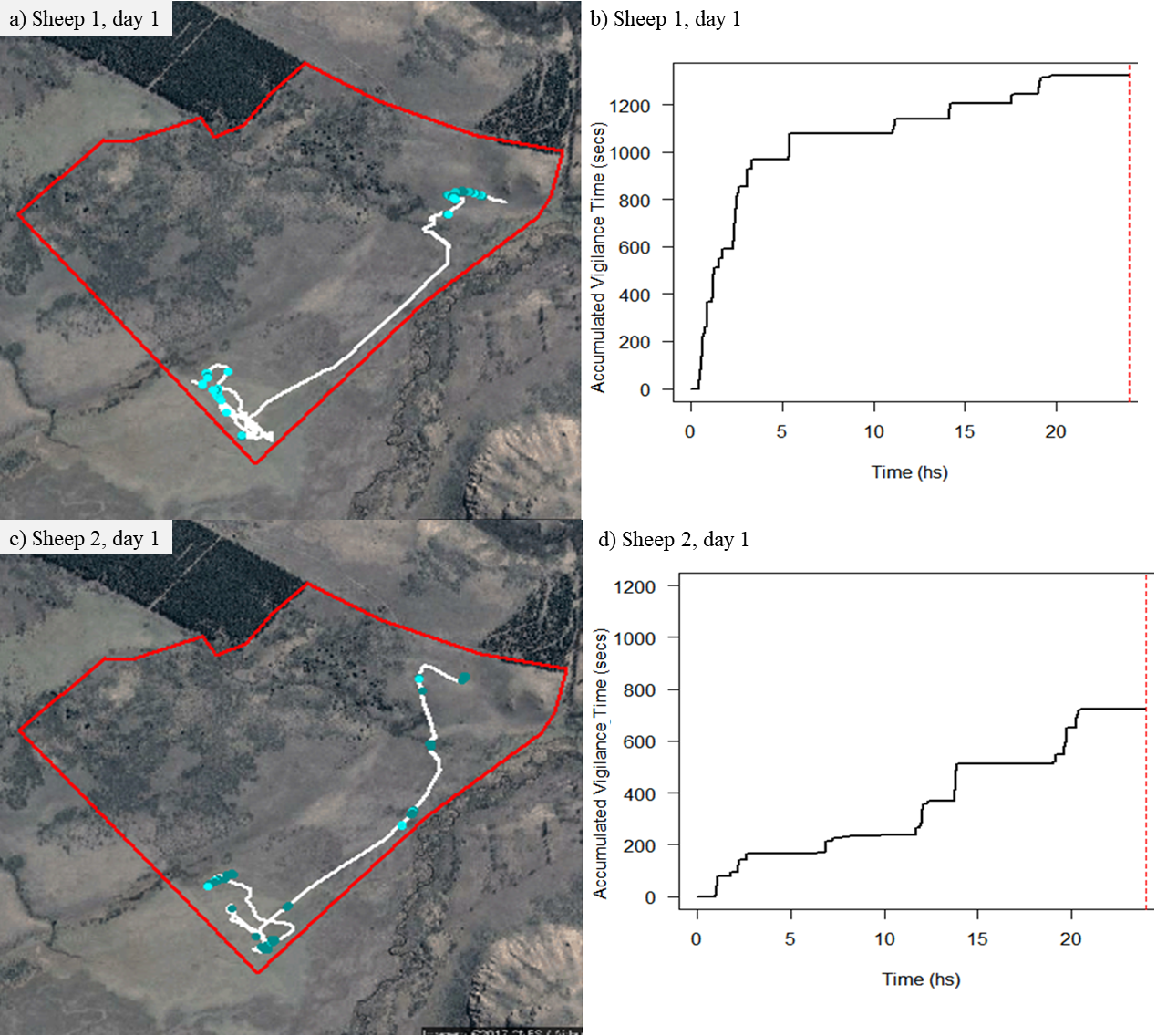


Figure S4.4.1: Movement path with vigilance rate (a and c) and accumulated vigilance interruptions through time (b and d) for sheep 1 and sheep 2, respectively on day 1.


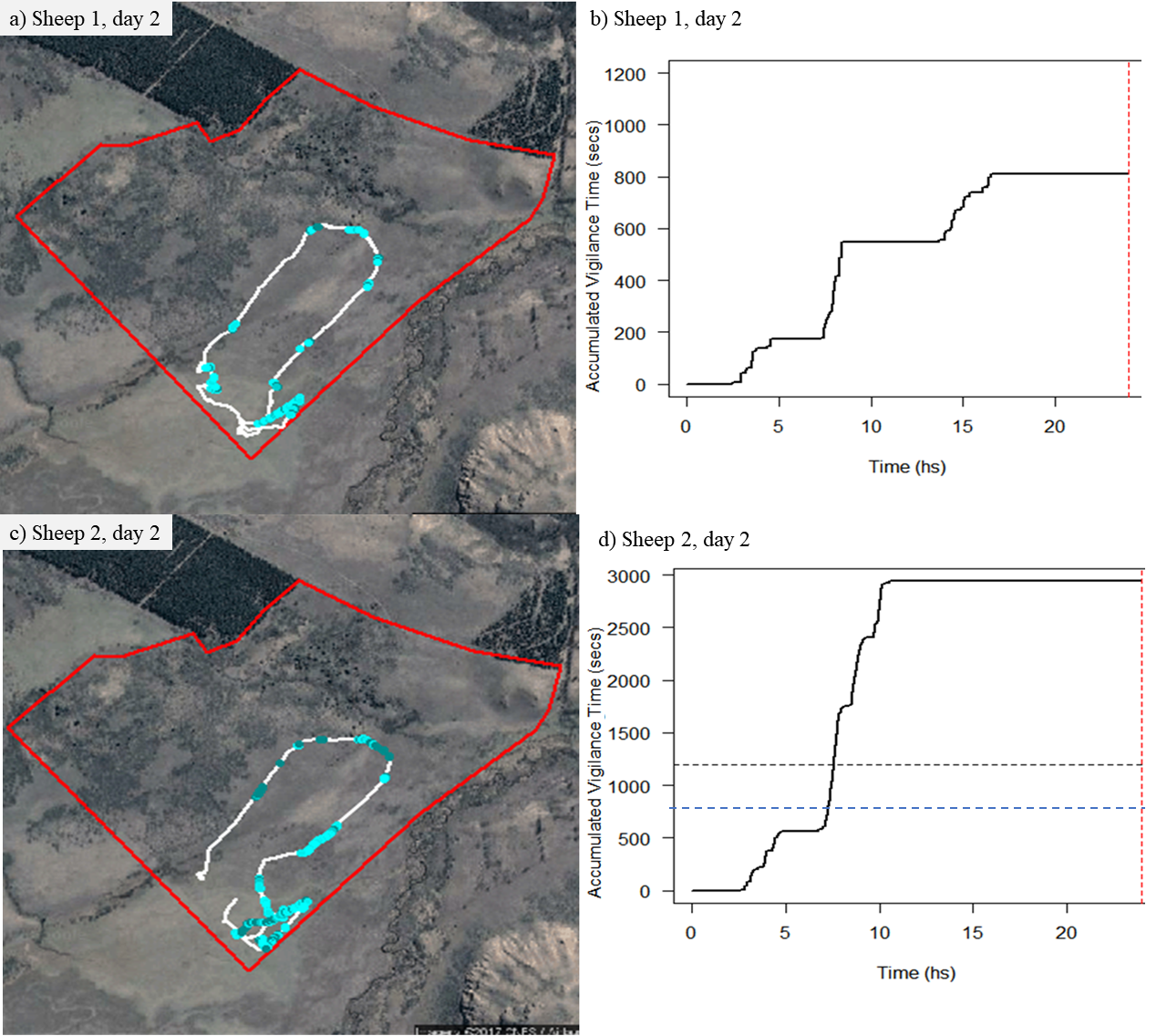


Figure S4.4.2: Movement path with vigilance rate (a and c) and accumulated vigilance interruptions through time (b and d) for sheep 1 and sheep 2, respectively on day 2.


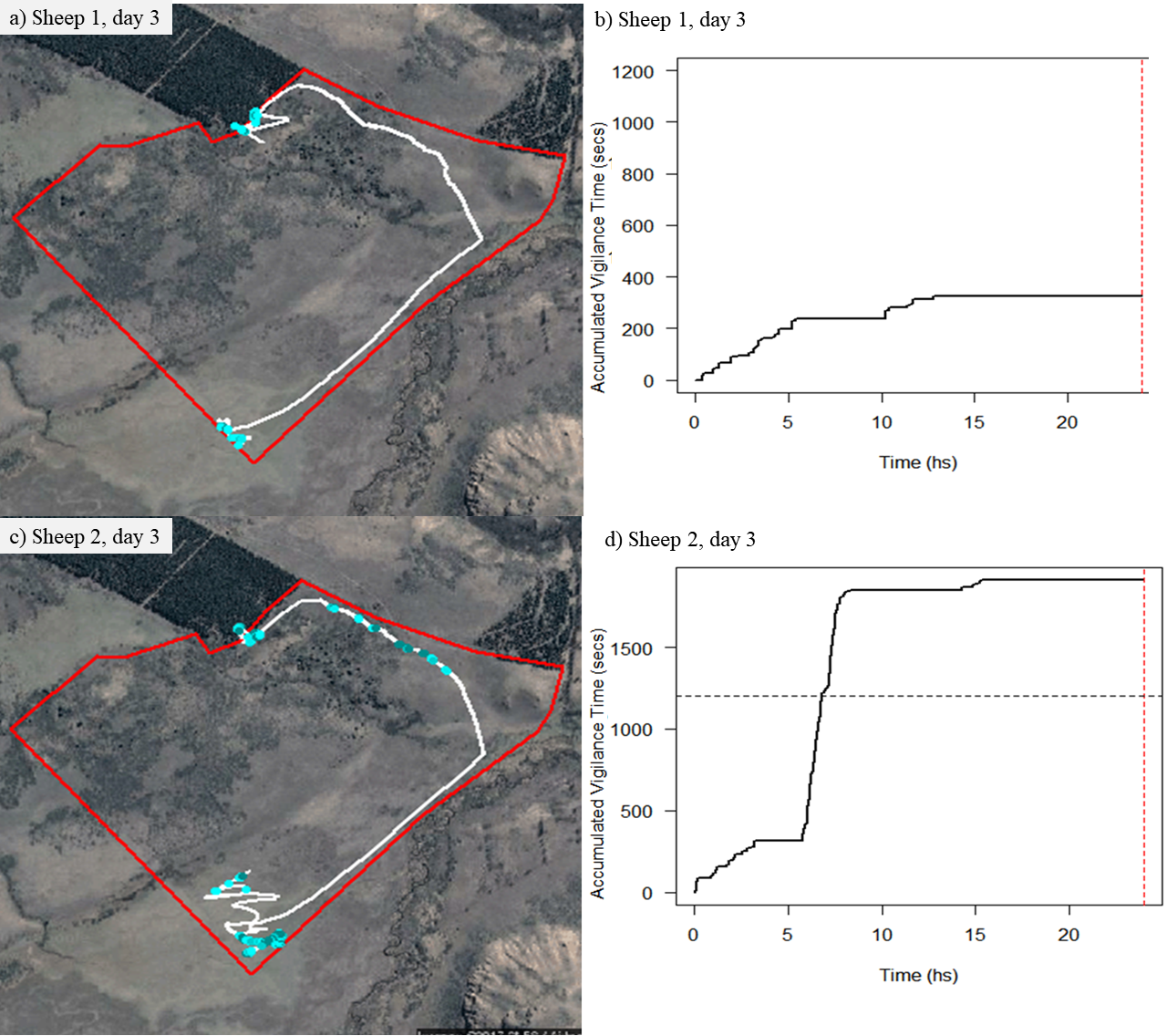


Figure S4.4.3: Movement path with vigilance rate (a and c) and accumulated vigilance interruptions through time (b and d) for sheep 1 and sheep 2, respectively on day 3.
